# Nutritional Status Changes During Pediatric Acute Lymphoblastic Leukemia Treatment: A Study from a Tertiary Care Hospital in Pakistan

**DOI:** 10.1177/11795565261454801

**Published:** 2026-06-10

**Authors:** Sadia Imran, Namrita Rai, Malick Muhammed Sabih Masood, Wasfa Farooq, Kishwer Nadeem

**Affiliations:** 1 Department of Pediatric Hematology & Oncology, Indus Hospital & Health Network, Karachi, Pakistan; 2 University of Eastern Finland, Kuopio, Northern Savo, Finland

**Keywords:** acute lymphoblastic leukemia, nutrition, malnutrition, Pakistan, pediatric

## Abstract

**Background:**

Acute Lymphoblastic Leukemia (ALL) is the most common pediatric cancer with a 5-year survival rate of 90% in High Income Countries (HICs), but in lower-to-middle-income countries (LMICs) survival rates fall below 60%. This is influenced by many factors; one of the major ones being malnutrition.

**Objectives:**

To ascertain the changes in and association of nutritional status in the treatment of pediatric ALL patients.

**Design:**

This article investigated the association between nutritional status in patients receiving ALL treatment, and morbidity and mortality. This is a retrospective study for the period of May 2022 to April 2023 including a total of 332 patients.

**Methods:**

The study used WHO defined weight-for-age and height-for-age for patients from 1 year of age up till the age of 5 years old, and BMI for age for patients over the age of 5 to assess nutritional status. Various disease and treatment variables were analyzed with patients’ nutritional statuses.

**Results:**

From the sample, more than one-third of patients (38.5%) were malnourished with the majority of them being underweight or severely underweight. Wilcoxon signed-rank test for paired values showed a *P*-value of .0177 (*z* = 2.373) when used to compare patient weight at initial assessment and weight after intensive phase of treatment, indicating a significant change in weight between both timepoints. Nutritional status was significantly associated with the number of patients with positive bacterial cultures (*P* = .01) and multiple hospital admissions (*P* = .004), but was not significantly associated with mortality (*P* > .05).

**Conclusion:**

Nutritional status changed significantly after intensive phase of treatment. Malnutrition in ALL patients was significantly associated with bacterial infections, however, it was not significantly correlated to patient mortality. Future prospective studies with a longer study period should be planned to follow patients compromised nutrition to assess long term survival.

**Registration:**

Not applicable.

## Background

Acute Lymphoblastic Leukemia (ALL) is the most common pediatric cancer and accounts for approximately 25% of 400 000 annually diagnosed cancer cases in children worldwide.^1^ In recent times, the 5-year survival rate for ALL has increased to as high as 90% in high-income countries (HICs) but in lower-to-middle-income countries (LMICs), many factors such as extreme poverty, lack of access to healthcare facilities, high infection rates, and malnutrition contribute to increased morbidity and lower survival rates falling below 60%.^2,3^

A thorough understanding of the nutritional status of pediatric leukemia patients in Pakistan is essential, as it can have profound implications for their overall health, response to treatment, and quality of life. In LMICs where more than 80% of pediatric cancer patients reside, malnutrition has been reported to affect as many as 90% of patients suffering from pediatric leukemia, as opposed to around 10% in HICs.^4,5^

According to the World Health Organisation (WHO), malnutrition is defined as “deficiencies or excesses in nutrient intake, imbalance of essential nutrients or impaired nutrient utilization.”^6^ In the pediatric age group, it is assessed using anthropometric measures such as weight-for-height, weight-for-age, body-mass index (BMI), mid-upper arm circumference (MUAC), and triceps skin fold thickness (TSFT).^7,8^

Malnutrition, which can be a pre-existing state or consequence of the disease and its treatment can affect prognosis and treatment plans with increased instances of complications such as febrile neutropenia, increased side effects of treatment, prolonged duration of treatment, increased cases of relapse, and higher mortality rates.^9^ However, there is a gap in evidence on the effect of malnutrition on ALL related complications, outcomes, and long-term survival in LMICs.

Over the years, there have been many studies attempting to understand the association between nutritional status and ALL patient outcomes and to establish a comprehensive guideline for nutritional interventions during different stages of ALL treatment.^10,11^ There still appear to be many gaps in the literature pertaining to the subject, especially in Pakistan, which ranked 102 out of 125 countries on the Global Hunger Index.^12^ This is alarming considering that undernourishment in the Pakistani population rose from 12.1% in 2015 to 18.5% in 2023.^12^

This study based in the Department of Pediatric Hematology/Oncology of Indus Hospital & Health Network (IHHN), one of the largest childhood cancer units in the country, aims to identify the associations of baseline nutritional status with treatment related toxicity in the induction and consolidation phases of treatment of pediatric patients diagnosed with ALL. It also aims to find if there is an association between nutritional status and mortality in these patients, and to identify if initial poor nutritional status worsened or improved in pediatric ALL patients at the end of intensive treatment, prior to the start of maintenance phase.

## Methods

A retrospective chart review was conducted between 1st May 2022 and 30th April 2023, for pediatric patients with ALL being treated at IHHN, Karachi, Pakistan. Data was collected for patients between the ages of 1 year and 16 years from the Hospital Management and Information System (HMIS). Patients with any diseases apart from ALL, below the age of 1 year, and above the age of 16 years were excluded from the study.

Anthropometric measures included weight, height, and age obtained from HMIS collected at diagnosis and again, after intensive phase of treatment and immediately prior to maintenance phase (pre-maintenance). Patients were classified according to their nutritional status as per WHO guidelines to establish a baseline against patient demographics. Boys and girls were classified separately using gender specific growth charts.^13-15^ Weight-for-age and height-for-age was used for patients from 1 year of age up till the age of 5 years old, and BMI-for-age was used for patients over the age of 5, for the purpose of nutritional status classification. These measurements were plotted on the WHO growth charts mentioned above, their Z-scores calculated, and nutritional status obtained and sorted into ordinal categories as per WHO (Table 1).

**Table 1.** Anthropometric Measures Used to Classify Children into Nutritional Categories Using Gender Specific Growth Charts As Per WHO.

Birth to 5 y (Weight-for-age, weight for length)	
Weight classification	Definition
Obesity	Weight-for-length/height or BMI-for-age > 3 standard deviations (SD)
Overweight	Weight-for-length/height or BMI-for-age > 2 SD
Normal weight	Weight-for-age between ⩽2 and ⩾−2 SD
Underweight (thinness)	Weight-for-age < −2 SD and ⩾−3 SD
Severely underweight (severe thinness)	Weight-for-age < −3 SD
Age 5 till 16 y (BMI-for-age)	
Obesity	BMI-for-age > 2 SD
Overweight	BMI-for-age > 1 SD
Normal weight	BMI-for-age < −2 SD and ⩾−2 SD
Underweight (thinness)	BMI-for-age < −2 SD and ⩾−3 SD
Severely underweight (severe thinness)	BMI-for-age < −3 SD

Abbreviations: BMI, Body Mass Index; SD, standard deviation.

Patients were treated as per our National Acute Lymphoblastic Leukemia (ALL) Protocol (PSPO ALL-2020) that is based on the COG ALL Regimens with some modifications. This approach maximizes therapeutic efficacy, and minimizes toxicity, incorporating a crucial 7-day Prednisolone pro-phase with intrathecal therapy. This step stabilizes co-morbidities and nutritional deficiencies while assessing Prednisolone sensitivity before intensive chemotherapy. During induction, standard risk (SR) patients are treated with a 3-drug induction regimen of Vincristine (VCR), Peg-asparaginase, and corticosteroids, while high risk (HR) patients additionally receive anthracycline therapy with Daunorubicin. Post-induction therapy is tailored based on initial risk stratification and end-of-induction MRD response, with SR patients typically receiving consolidation therapy of 3 doses of Weekly IV Vincristine and Oral Mercaptopurine daily (28 days duration), and HR patients receiving a more intensive regimen with High Risk Consolidation (Modified BFM Consolidation) that includes Cyclophosphamide, Cytarabine, Peg-asparaginase, vincristine and oral mercaptopurine. Following consolidation, HR patients enter an Interim Maintenance phase with the Capizzi regimen, and SR patients received IM phase with non-escalating methotrexate followed by Delayed Intensification (DI) phase featuring Vincristine, anthracyclines, asparaginase, and dexamethasone. Intrathecal therapy is integrated throughout the treatment phases. After this intensive therapy, a maintenance phase of 2 years begins.

### Statistical Analyses

**Figure 1.**
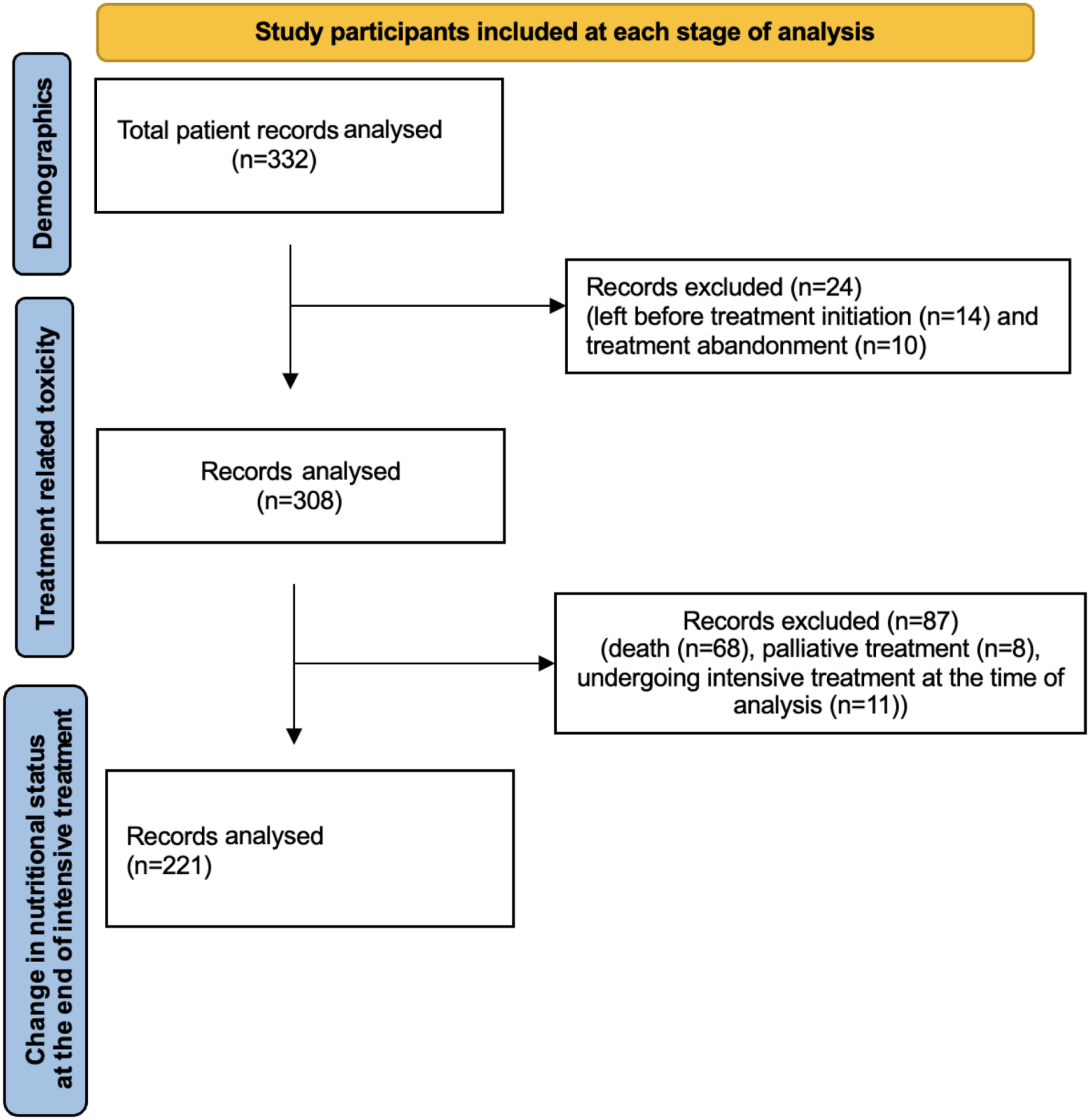
Number of patients included in each stage of analysis.

Descriptive statistics were applied to disease variables to identify associations with nutritional status using SPSS software (version 26). Statistical significance was considered at a cut off of *P*-value <.05. For tests that showed significant results, odds ratio was calculated to ascertain whether singular exposures lead to an increased outcome. The analysis was conducted in 3 steps. The first was to analyze whether demographic or other patient variables were associated with nutritional status. The second was to ascertain whether nutritional status was associated with treatment related toxicity and short-term patient outcomes over the span of the study period. Lastly patient nutritional status was analyzed to ascertain whether significant changes were observed prior to maintenance phase as compared to initial nutritional status at diagnosis. All patients were included in the first step of analysis (n = 332), patients who left before treatment and those who abandoned treatment were excluded for the second step evaluating treatment related toxicity (n = 308 remaining). In the final step, only patients who reached maintenance phase of treatment were included (n = 221; Figure 1).

OpenEpi version 3 was used to perform a power analysis to justify the final sample size of n = 221. The exposed group was defined as the undernourished group at diagnosis (n = 75) which comprised of patients who were underweight and severely underweight. The unexposed group comprised of patients with normal nutritional status at diagnosis (n = 138). The outcome of interest was worsened nutritional status at pre-maintenance with an observed risk of 53.3% amongst undernourished patients and 27.5% in normal nutritional status patients at diagnosis. At a two-sided confidence level of 95% the study sample provided 96.68% power. This exceeds the traditional expectation of 80% statistical power and proves that a sample size of 221 was more than sufficient in deriving clinically relevant results.

## Results

A total of 332 patients were included with a 1.6:1 male to female ratio. A majority of patients (41.3%) were between the ages of 5 to 10 years, followed by children aged 1 to 4 years, and 11 to 16 years (Table 2). Most patients were from Karachi and interior Sindh, followed by Balochistan, and some from other regions such as Punjab, Khyber Pakhtunkhwa (KP), and Afghanistan. Around two-thirds of patients (n = 204, 61.5%) at presentation had normal nutritional status. More than one-third of patients (n = 128, 38.5%) were malnourished with the majority of them being underweight or severely underweight. A significant association was found between patients’ age group and nutritional status. The oldest age group of 11 to 15 years had significantly fewer normal weight patients and significantly higher overweight patients than expected (*P* = .03). In contrast, the younger age groups (1-5 and 6-10) did not show significant deviations from the expected nutrition status. Gender and region were not significantly correlated with nutritional status (Table 3).

**Table 2.** Patient Demographics Including Age, Gender and Area of Residence.

Demographic variables		Number of patients	
		Total (n = 332)	Percentage of patients
Gender	Male	205	61.7
	Female	127	38.3
Age	1-4	112	33.7
	5-10	137	41.3
	11-16	83	25.0
Region	Afghanistan	20	6.0
	Balochistan	65	19.6
	Interior Sindh	117	35.2
	Karachi	122	36.7
	KP	3	0.9
	Punjab	5	1.5
Risk level	High risk	217	65.4
	Standard risk	115	34.6

Abbreviations: KP, province of Khyber Pakhtunkhwa in Pakistan.

**Table 3.** Demographic Variables Associated with Nutritional Status. Changes in Initial and Pre-Maintenance Nutritional Status.

	Nutritional status						
Age	Obese	Overweight	Normal	Underweight	Severely underweight	Total	*P*-value
1-5	0	2	95	29	17	143	.02
6-10	1	0	71	17	17	106	
11-15	1	6	38	19	19	83	
Total	2	8	204	65	53	332	
	Nutritional status						
Gender	Obese	Overweight	Normal	Underweight	Severely underweight	Total	*P*-value
Male	2	6	121	37	39	205	.30
Female	0	2	83	28	14	127	
Total	2	8	204	65	53	332	
	Nutritional status						
Region	Obese	Overweight	Normal	Underweight	Severely underweight	Total	*P*-value
Afghanistan	0	0	16	3	1	20	.24
Balochistan	0	2	46	8	9	65	
Interior Sindh	0	2	66	25	24	117	
Karachi	1	3	70	29	19	122	
Khyber Pakhtunkhwa and Punjab	1	1	6	0	0	8	
Total	2	8	204	65	53	332	
	Nutritional status – prior to maintenance phase						
Nutritional status – at diagnosis	Obese	Overweight	Normal	Underweight	Severely underweight	Total	*P*-value
Obese	0	1	1	0	0	2	<.001
Overweight	2	1	3	0	0	6	
Normal	0	1	99	21	17	138	
Underweight	0	0	10	13	17	40	
Severely underweight	0	0	4	8	23	35	
Total	2	3	117	42	57	221	

Patients were stratified as high risk (65.4%) and standard risk (34.6%). Standard risk patients were those with B-cell precursor ALL who were between 1 and 10 years of age and who had a leukocyte count of <50 × 10^9^/L. High risk patients were those who did not meet the criteria for standard risk, all cases of T-cell ALL, post induction MRD positive that is, ⩾0.01%. Any patient who had (a) CNS-3 status; (b) overt testicular leukemia; (c) adverse genetic features like *t*(9;22) BCR-ABL1 fusion, rearranged MLL or hypodiploidy < 45 chromosome; or (d) poor early response (defined as ⩾1000 absolute blast count at day 8 CBC), was also classified as high risk.

At the time of analysis, 221 patients out of 332 (66.6%) reached maintenance stage of treatment, 68 patients had died (20.5%), while the remaining had either abandoned treatment (n = 10, 3.0%), left before treatment initiation (n = 14, 4.2%), were undergoing palliative treatment (n = 8, 2.4%), or were still undergoing intensive treatment (n = 11, 3.3%; Figure 1). Infection was the most common cause of mortality (n = 56, 82.4%), followed by disease progression (n = 6, 8.8%), hemorrhage (n = 4, 5.9%), and drug toxicity (n = 2, 2.9%). The most commonly acquired infection leading to mortality was Carbapenem-resistant Enterobacterale infection (n = 22, 39.3%). Nutritional status was not significantly associated with mortality (*P* = .9).

In the patients who completed the intensive phase of treatment and reached maintenance phase, nutritional status significantly changed post intensive phase of treatment (*P* < .001; Table 3). A Wilcoxon signed-rank test for paired values was used to compare nutritional measurements at initial assessment and nutritional measurements prior to maintenance phase. A *P*-value of .0177 (corresponding to a change in *z*-score = −2.373) was observed, indicating a significant change in weight between both timepoints. The odds that a patient experienced negative changes in nutritional status (with or without changes in nutritional status category) following intensive phase of treatment were 0.31 (OR 0.31, 95% CI 0.13-7.04, *P* = .005). Odds ratio was then calculated separately for those patients who had improved nutritional status at premaintenance, and worsened nutritional status at premaintenance, keeping various exposure variables in mind. While associations with age, disease type (B-ALL or T-ALL), risk status, and infection did not reach significance, gender showed a significant result with worsened nutritional status post-intensive phase (*P* = .03). When stratified by gender, it was found that male patients with malnutrition were 2.7 times more likely than female patients to experience significant negative changes in nutritional status following intensive phase of treatment (OR 2.68, 95% CI 1.12-6.39, *P* = .03).

To evaluate the association of nutritional status with treatment related toxicity, the number of hospital admissions and positive bacterial cultures were taken into account. (Table 4). Nutritional status was significantly correlated to the number of hospital admissions (*P* = .004) and the number of positive bacterial cultures in patients (*P* = .01). When the same indicators were compared amongst children within the same risk group, that is, SR and HR, these tests were not statistically significant.

**Table 4.** Nutritional Status Associated with Treatment Related Toxicity.

	Number of hospital admissions						
Nutritional status	0	1-5	6-10	11-15	>15	Total	*P*-value
Obese	0	2	0	0	0	2	.004
Overweight	0	7	1	0	0	8	
Normal	2	147	38	5	1	193	
Underweight	0	47	9	1	1	58	
Severely underweight	0	40	6	1	0	47	
Total	2	243	54	7	2	308	
	Number of positive cultures						
Nutritional status	0	1-5	6-10	>10	Total		*P*-value
Obese	1	1	0	0	2		0.01
Overweight	3	5	0	0	8		
Normal	97	87	8	1	193		
Underweight	32	23	3	0	58		
Severely underweight	22	22	3	0	47		
Total	155	138	14	1	308		

## Discussion

For the purpose of our study, any nutritional status other than normal was considered to be an indicator of malnutrition. A total of 38.6% of patients were found to be malnourished at the time of diagnosis, similar to a study conducted in India, in which the prevalence of malnutrition was 44.0%.^16^

Nutritional status was statistically significant when cross tabulated with age in ALL patients, which is consistent with previous literature in which age is independently associated with nutritional status.^17^ It is also worth investigating whether this correlation is attributed to the anthropometric measurements used to evaluate nutritional status. When using different anthropometric measures, variable results for malnutrition have been found in previous studies.^8,18^ Nutritional status was not significantly correlated to the gender of the patient or region of residence.

Overall patient nutritional status changed significantly after intensive phase of treatment, in comparison to nutritional status at diagnosis (*P* < .001). This may be attributed to the rapid loss of lean body mass during treatment regimens given during induction^19^ and consolidation phases of treatment. This finding supports the need for dedicated, individualized, and focused nutrition plans during the intensive phase of ALL treatment in LMICs.

All patients were given nutritional interventions according to the same guidelines. All patients were thoroughly counseled to follow a nutrient dense oral diet. Patients under the median weight, or taking an insufficient oral diet indicating a moderate nutritional risk were given oral supplements along with a nutrient-dense oral diet. Hygienic preparation guidelines were advised. Enteral nutrition was provided to patients who were severely underweight and/or to patients consuming less than 50% of their required daily calories for over 3 days, if the patient and their guardian consented to it. In cases where consent was obtained and enteral nutrition was not possible, parenteral nutrition was advised.

We analyzed the predisposition of factors such as age group, gender, diagnosis, risk group, positive cultures, and number of hospital admissions on the likelihood of improved nutritional status, and worsening nutritional status. Of all the mentioned variables, only gender was found to have a significant association with worsened nutritional status at premaintenance. Male patients with malnutrition were 2.7 times more likely to experience worsened nutritional status following intensive phase of treatment (OR 2.68, 95% CI 1.12-6.39, *P* = .03) as compared to female patients. Prior studies evaluating nutritional status changes in ALL patients following induction phase of treatment or otherwise have not evaluated gender as an independent variable affecting the outcome. However, 1 study found a link between gender and acquiring infections. It reported a sixfold increased risk of male children with ALL acquiring infection during treatment (OR 5.95, 95% CI 1.29-26.43; *P* = 0.03).^18^ The predisposition to treatment related toxicity such as infection and worsening nutritional status should be investigated so as to be able to provide timely interventions and prevent toxicity. Risk status was not found to have a significant correlation with nutritional status change post intensive phase of treatment, which is in stark contrast to prior studies in which strong associations have been found.^19^

Nutritional status was not significantly associated with patient mortality (*P* > .05). This is consistent with previous studies which do not show a significant link between malnutrition at diagnosis and mortality.^8^ However, our results may be limited by relatively fewer number of patients in the overweight and obese nutritional categories. It is generally very rare to see overweight and obese patients at IHHN. This may be attributed to the fact that IHHN is a completely free-of-cost welfare hospital in an LMIC, and most of the patients seeking treatment in the center arrive from socioeconomically disadvantaged backgrounds.

To evaluate the correlation between nutritional status of ALL patients and a predisposition to infections, the number of hospital admissions for conditions apart from routine workup, and positive bacterial cultures were taken into account. Nutritional status was found to have a significant association with both, the number of hospital admissions (*P* = .004), and the number of positive bacterial cultures (*P* = .01). This result is similar to a study linking malnutrition to severe infections in pediatric cancer patients.^20^ Due to the nature of this study being retrospective, serum albumin could not be taken into account since this test is conducted at various phases of treatment, instead of at fixed times prior to treatment initiation and prior to maintenance phase.

In the future, a prospective study should be planned over a longer study period to conduct a more in-depth multivariate analysis of the association of malnutrition at diagnosis in ALL patients and long-term disease outcomes. For a future prospective study, serum albumin values should be acquired at the time of diagnosis and the association thoroughly investigated.

## Conclusion

Malnutrition in ALL patients was significantly associated with bacterial infections. Male patients with malnutrition at diagnosis were 2.7 times more likely to experience significantly worsened nutritional status at the end of intensive phase of treatment. While malnutrition was not significantly correlated to patient mortality, future prospective studies with a longer study period should be planned to follow patients with malnutrition or compromised nutrition for long-term survival.
